# Single Versus Dual Antiplatelet Therapy After Transcatheter Aortic Valve Implantation in Patients Without Chronic Anticoagulation

**DOI:** 10.3390/jcm15114381

**Published:** 2026-06-05

**Authors:** Monirah A. Albabtain, Aisha Alrasheedi, Razan M. Awan, Maha Alharthi, Zaid Alanazi, Nawaf Aldhubayti, Amr A. Arafat

**Affiliations:** 1 Research Department, Prince Sultan Cardiac Center, Riyadh 12233, Saudi Arabia; malbabtain@pscc.med.sa; 2 Pharmacy Department, Prince Sultan Cardiac Center, Riyadh 12231, Saudi Arabia; mmharthi.9@gmail.com (M.A.); zalanazi@pscc.med.sa (Z.A.); nalmitiri@pscc.med.sa (N.A.); 3 Pharmacy Department, King Fahad Specialist Hospital, Buraydah 52366, Saudi Arabia; aishamutie@gmail.com; 4 Pharmacy Department, King Fahad Armed Forces Hospital, Jeddah 23311, Saudi Arabia; razan.awan@gmail.com; 5 Research & Innovation Institute, Ministry of Defense Health Services, Riyadh 12426, Saudi Arabia

**Keywords:** transcatheter aortic valve implantation, antiplatelet therapy, aspirin, clopidogrel, time-varying Cox, inverse-probability-of-treatment weighting

## Abstract

**Background:** The optimal antiplatelet strategy after transcatheter aortic valve implantation (TAVI) in patients who do not require long-term oral anticoagulation remains debated. Randomized trial evidence supports single antiplatelet therapy (SAPT) over dual antiplatelet therapy (DAPT), yet real-world practice patterns and the magnitude of benefit in contemporary TAVI populations remain heterogeneous. **Methods:** We analyzed consecutive patients undergoing TAVI at a single tertiary center between April 2009 and April 2023 who were discharged on an antiplatelet regimen only. Patients on chronic oral anticoagulation were excluded. The exposure was defined by the discharge regimen (SAPT vs. DAPT), treated as a time-varying variable with person-time split at the documented date of any regimen change. The pre-specified efficacy endpoint was ischemic major adverse cardiovascular events (iMACE: death, stroke, or myocardial infarction); net adverse clinical events (NACEs) added major bleeding. The primary analysis was a time-varying Cox model adjusted for baseline variables. Sensitivity analyses included an intention-to-treat Cox model, a 6-month landmark Cox model, and an inverse-probability-of-treatment-weighted (IPTW) time-varying Cox model. **Results:** Of 662 eligible patients, 147 (22.2%) were discharged on SAPT and 515 (77.8%) on DAPT. Median follow-up was 34 months (IQR 14–52). During follow-up, 141 iMACE and 146 NACEs occurred. In the primary time-varying Cox model, the adjusted hazard ratio for DAPT versus SAPT was 1.28 (95% CI 0.81–2.04; *p* = 0.292) for iMACE and 0.71 (95% CI 0.44–1.14; *p* = 0.159) for NACE. None of the sensitivity models demonstrated a statistically significant difference between groups. Major bleeding was rare (six events; two SAPT, four DAPT). The 30-day landmark analysis showed no signal of an effect of regimen on late stroke (HR 1.12, 95% CI 0.39–3.12). **Conclusions:** In a contemporary real-world TAVI cohort, no statistically significant difference between SAPT and DAPT was observed for ischemic or net adverse clinical events. These findings demonstrate no ischemic disadvantage of SAPT compared with DAPT in real-world practice and are consistent with the randomized evidence base supporting SAPT as a reasonable default antiplatelet strategy after TAVI in patients without another antithrombotic indication. The bleeding endpoint was underpowered, and the expected bleeding advantage of SAPT could not be independently evaluated in this cohort.

## 1. Introduction

Transcatheter aortic valve implantation (TAVI) has evolved from a niche intervention reserved for inoperable patients to a first-line treatment for severe symptomatic aortic stenosis across all surgical-risk strata [1,2]. Contemporary international guidelines from the European Society of Cardiology/European Association for Cardio-Thoracic Surgery and the American College of Cardiology/American Heart Association now place TAVI on an equal footing with surgical aortic-valve replacement for most elderly patients and for many younger patients at intermediate or high surgical risk [3,4].

Despite this procedural maturity, the optimal antithrombotic strategy after TAVI has remained unsettled for more than a decade. Early practice was extrapolated from coronary stenting and favored a short course of dual antiplatelet therapy (DAPT) in the belief that it would reduce early thromboembolic events and prevent valve-leaflet thrombosis [5,6,7,8]. That empirical choice was challenged by the ARTE and POPular TAVI trials, which showed that single antiplatelet therapy (SAPT) was associated with less bleeding and no increase in ischemic events compared with DAPT in anticoagulant-free patients [9,10]. Trials of added oral anticoagulation (GALILEO with rivaroxaban and ATLANTIS with apixaban) further clarified that routine anticoagulation is harmful in the absence of a specific indication [11,12]. Subsequent meta-analyses reinforced the SAPT-favorable message, and the 2020 ACC/AHA and 2021 ESC/EACTS valvular guidelines now recommend lifelong aspirin monotherapy as the default antiplatelet strategy after TAVI in patients without another indication for antithrombotic drugs [3,4,13].

Randomized trial populations, however, are narrower than the contemporary real-world TAVI population, which has progressively expanded to include older, lower-risk, and more comorbid patients [14]. Observational confirmation of the randomized findings in unselected cohorts remains important, particularly when a non-trivial proportion of patients have their antiplatelet regimen modified during follow-up, a feature that challenges conventional intention-to-treat Cox modeling and calls for methods that accommodate time-varying exposure.

Accordingly, we examined the effect of discharge antiplatelet strategy (SAPT vs. DAPT) on ischemic and net clinical outcomes in a consecutive TAVI cohort at a tertiary center, using an analytic hierarchy that placed a time-varying Cox model at the center of the primary analysis and incorporated complementary sensitivity analyses (intention-to-treat, 6-month landmark, and inverse-probability-of-treatment weighting) to address the most important sources of bias in observational comparisons of antithrombotic therapy.

## 2. Methods

### 2.1. Study Design

This was a single-center, retrospective, observational cohort study reported in accordance with the Strengthening the Reporting of Observational Studies in Epidemiology (STROBE) statement for cohort studies [15]. Consecutive patients undergoing TAVI between April 2009 and April 2023 were enrolled in a prospectively maintained institutional registry and formed the source population for the present analysis. The date of the last clinical follow-up contributing to this report was April 2025.

The study protocol was reviewed and approved by the institutional Research Ethics Committee (Approval # 1679). The requirement for individual patient consent was waived on the basis of the retrospective design and the use of de-identified clinical data. The study was performed in accordance with the principles of the Declaration of Helsinki [16].

### 2.2. Participants

#### 2.2.1. Eligibility Criteria

All consecutive adult patients (age ≥ 18 years) who underwent TAVI at the center during the study period and who survived to hospital discharge with documentation of a defined antithrombotic regimen at discharge were eligible. Eligible procedures included first-time native-valve TAVI as well as valve-in-valve TAVI performed through any access route (transfemoral, transapical, transaortic, transaxillary, or transcaval). Patients were included irrespective of surgical risk category, symptom class, or left-ventricular function.

#### 2.2.2. Exclusion Criteria

Patients receiving chronic oral anticoagulation (OAC) with warfarin or a direct oral anticoagulant (DOAC) at discharge were excluded from the primary analytic cohort. This restriction mirrors the design of the POPular-TAVI Cohort A trial and isolates the clinical question to patients for whom a pure antiplatelet decision is actually being made [9]; that is, the subgroup of contemporary TAVI patients without a competing indication for chronic anticoagulation. Patients with procedural death, in-hospital death before discharge, or no documented discharge antithrombotic regimen were also excluded.

### 2.3. Variables

#### 2.3.1. Exposure

The exposure of interest was the antiplatelet regimen at discharge, classified as single antiplatelet therapy (SAPT; aspirin or a P2Y12 inhibitor alone) or dual antiplatelet therapy (DAPT; aspirin plus a P2Y12 inhibitor, most commonly clopidogrel). Within the SAPT group, 92 of 147 patients (62.6%) received aspirin monotherapy, and 55 (37.4%) received P2Y12-inhibitor monotherapy (predominantly clopidogrel; one patient on ticagrelor). Within the DAPT group, the combination was aspirin plus clopidogrel in 514 of 515 patients (99.8%) and aspirin plus ticagrelor in one patient; no patient was discharged on prasugrel. The regimen was extracted from the hospital-discharge prescription and verified against the first post-discharge clinic visit. Because a non-trivial fraction of patients had their antiplatelet regimen changed during follow-up (typically DAPT de-escalated to SAPT after the conventional 3–6-month post-TAVI window), we recorded for every patient the date and nature of any regimen change, taken from outpatient pharmacy records and clinic visits. Patients who never changed regimen contributed a single exposure interval; patients who changed regimen contributed two consecutive exposure intervals.

#### 2.3.2. Outcomes

The efficacy primary endpoint was ischemic major adverse cardiovascular events (iMACEs), a composite of all-cause mortality, any stroke, or myocardial infarction, whichever occurred first. The secondary endpoint was net adverse clinical events (NACEs), a composite of iMACE plus major bleeding. Major bleeding was also reported as an isolated safety endpoint. Individual components (all-cause death, any stroke, myocardial infarction, major bleeding) were analyzed as secondary endpoints with death as a competing risk where applicable.

All outcomes were adjudicated against the Valve Academic Research Consortium 3 (VARC-3) definitions. Stroke included both ischemic and hemorrhagic events, confirmed by imaging and by neurology consultation [17]. Myocardial infarction was defined according to the Fourth Universal Definition [18]. Major bleeding corresponded to VARC-3 types 3b and 4 (life-threatening or fatal). Bleeding events were ascertained through four complementary channels. First, the institutional TAVI registry captures every re-admission to the index center, including admissions with a bleeding diagnosis. Second, the index center has shared electronic health records with other facilities. Third, every patient in the cohort had scheduled outpatient follow-up at the index center (typically at months 1, 6, and 12 and annually thereafter, with additional unscheduled visits where clinically indicated); the structural-heart-disease team elicited and documented any interim bleeding episode at each visit, including episodes managed at outside facilities. Fourth, the bleeding endpoint used the VARC-3 definition restricted to types 3b and 4, life-threatening or fatal bleeding, which is the most stringent end of the VARC-3 spectrum. Ischemic events (death, stroke, myocardial infarction) were identified from the same registry sources; source documents (discharge summaries, imaging reports, death certificates) were re-reviewed for every candidate event. A new permanent pacemaker was implanted post-procedurally in 71 of 662 patients (10.7%).

#### 2.3.3. Covariates

Baseline characteristics were recorded at the time of the pre-procedural work-up. Demographic variables included age in years and sex. Anthropometric and laboratory variables included body-mass index (BMI, kg/m^2^), hemoglobin (g/dL), platelet count (×10^9^/L), and serum creatinine (µmol/L) from the closest pre-procedural blood sample. Echocardiographic left-ventricular ejection fraction (LVEF, %) was taken from the most recent pre-procedural transthoracic study.

Cardiovascular comorbidities were abstracted as binary variables: hypertension, diabetes mellitus, dyslipidemia, history of prior myocardial infarction, prior percutaneous coronary intervention, prior cardiac surgery (including prior aortic-valve replacement), prior stroke or transient ischemic attack, carotid artery stenosis, pre-procedural permanent pacemaker, and pre-procedural implantable cardioverter-defibrillator. Non-cardiovascular comorbidities included chronic lung disease, home oxygen therapy, chronic liver disease, and chronic kidney disease, either dialysis-dependent or not on dialysis.

### 2.4. Data Sources

All clinical, procedural, and outcome data were extracted from the institutional TAVI registry, which is populated prospectively by the structural-heart-disease clinical team and updated after every scheduled outpatient visit (typically 1, 6, and 12 months post-procedure and annually thereafter). Source documents accessed for verification included pre-procedural clinic notes, echocardiographic reports, catheterization-laboratory procedure reports, hospital discharge summaries, laboratory results, and outpatient clinic reports.

### 2.5. Potential Sources of Bias and Measures to Minimize Them

Several sources of bias are inherent to an observational cohort study of antiplatelet therapy after TAVI. First, allocation to SAPT versus DAPT was at the clinician discretion and was expected to be associated with patient frailty, anticipated bleeding risk, and coronary-disease burden; residual confounding by indication was the principal threat to causal interpretation. We addressed measured confounding through multivariable Cox adjustment for a parsimonious confounder set and through inverse-probability-of-treatment weighting (IPTW) as a robustness analysis. Unmeasured confounding (particularly by frailty) could not be eliminated but was mitigated by including clinically relevant proxies (age, chronic kidney disease, chronic lung disease, LVEF).

Second, because patients changed antiplatelet regimen during follow-up (typically DAPT to SAPT at or after the 3–6-month mark), a strict intention-to-treat Cox model anchored to the discharge regimen would misclassify a substantial fraction of person-time. We therefore pre-specified a time-varying Cox model, with person-time split at the documented date of regimen change, as the primary analysis. The intention-to-treat Cox was retained as a sensitivity analysis.

Third, because bleeding itself commonly triggers de-escalation of antiplatelet therapy, time-varying analysis of the bleeding endpoint is vulnerable to reverse causation (the event causes the exposure change rather than the other way round). For this reason, the bleeding endpoint was analyzed in an intention-to-treat framework only and reported descriptively, with explicit acknowledgement of the direction of the residual bias.

Fourth, the Kaplan–Meier estimator assumes that the cohort can be partitioned into fixed, mutually exclusive risk sets for the exposure of interest, an assumption that is mathematically violated when the exposure changes during follow-up. There is no Kaplan–Meier analog of the time-varying Cox estimator. The Kaplan–Meier estimates of freedom from iMACE and NACE are therefore drawn by baseline regimen and correspond to the intention-to-treat sensitivity analysis rather than to the primary time-varying analysis.

Fifth, peri-procedural stroke within 30 days of TAVI is largely driven by procedural embolic phenomena and is unlikely to be modulated by maintenance antiplatelet choice. To avoid diluting any late effect with the peri-procedural signal, a 30-day landmark analysis for stroke was pre-specified.

Finally, outcome ascertainment bias was minimized by the prospective nature of the registry and by re-adjudication of every candidate event against source documents. Patients with no recorded follow-up after discharge were excluded from time-to-event analyses to avoid zero-follow-up distortions.

### 2.6. Study Size

No a priori sample-size calculation was performed; the analytic cohort comprised all eligible consecutive patients. The observed event count in the primary cohort (approximately 141 iMACE events over a median follow-up of 34 months) supports a parsimoniously adjusted multivariable Cox model with approximately ten candidate covariates, observing the conventional events-per-variable guideline of ≥10. The number of bleeding events was anticipated to be small after exclusion of anticoagulated patients, and bleeding was accordingly pre-specified as a descriptive rather than inferential endpoint.

### 2.7. Statistical Methods

#### 2.7.1. Descriptive Analysis

Continuous variables were summarized as median (interquartile range, IQR) and compared with the Mann–Whitney U test; categorical variables were summarized as n/N (%) and compared with the χ^2^ test, or Fisher’s exact test when any expected cell count was below five. All tests were two-sided with a nominal significance level of 0.05. Continuous variables were entered into multivariable models as linear terms; no categorization was performed [19].

#### 2.7.2. Primary and Sensitivity Analyses

The primary analysis was a Cox proportional-hazards regression of the hazard of each composite endpoint on the antiplatelet regimen treated as a time-varying covariate, with person-time partitioned at the documented date of regimen change in a counting-process “start, stop” data structure [20]. Robust standard errors clustered by patient identifier were used throughout. Three sensitivity analyses were performed: (i) an intention-to-treat Cox model using the baseline regimen as a fixed exposure; (ii) a 6-month landmark Cox model in patients event-free at 6 months with the follow-up clock reset to the landmark [21]; and (iii) an IPTW time-varying Cox model using stabilized weights derived from a logistic regression of baseline DAPT-versus-SAPT on the same covariate set, trimmed at the 1st and 99th percentiles [22]. Balance was assessed through absolute standardized mean differences (|SMD|) with a threshold of 0.10 for adequate balance. For non-fatal components, death was treated as a competing event; cause-specific hazards were estimated by Cox regression in which competing deaths were censored [23].

To quantify the potential influence of unmeasured confounding on each effect estimate, E-values were computed for the point estimate and for the confidence-interval limit closest to the null, using the approach of VanderWeele and Ding [24].

Procedural era, dichotomized as pre-2020 versus 2020–2023 to align with the publication of POPular TAVI and the 2021 ESC/EACTS valvular guidelines [3,9], was added to the adjusted covariate set in all multivariable Cox models. A formal test for effect modification by era was performed by adding an era × regimen interaction term to the primary time-varying Cox model, and era-stratified hazard ratios were obtained from the interaction model and from separately fitted era-restricted Cox models.

#### 2.7.3. Missing Data and Software

Missing data were handled by complete-case analysis at the patient level, with no missingness-based exclusion of covariates. Each variable was summarized on its complete cases, and the denominator was reported explicitly on the row. For the multivariable time-varying Cox, intention-to-treat Cox, and IPTW models, an adjusted analysis was fit on the 571 patients who had non-missing data on all twelve pre-specified covariates and on follow-up time. The 6-month landmark model was fit on the 532 patients who were event-free at the 6-month landmark. No imputation was performed. All analyses were performed in Stata version 18 (StataCorp LLC, College Station, TX, USA).

## 3. Results

### 3.1. Cohort and Follow-Up

A total of 662 patients met the eligibility criteria for the primary antiplatelet-only cohort, of whom 147 (22.2%) were discharged on SAPT and 515 (77.8%) on DAPT. The median follow-up was 34 months ([IQR] 14–52). A documented change in antiplatelet regimen occurred in a substantial minority of patients, predominantly a DAPT-to-SAPT de-escalation at or after the conventional 3- to 6-month window.

### 3.2. Baseline Characteristics

Baseline clinical characteristics of the SAPT and DAPT groups are presented in Table 1. SAPT-treated patients were slightly younger and leaner than DAPT-treated patients and had a substantially lower prevalence of prior percutaneous coronary intervention (13.7% vs. 36.9%; *p* < 0.001), consistent with indication-based prescribing. The two groups were otherwise similar with respect to sex distribution, LVEF, routine laboratory parameters, and the remaining comorbidities.

A strong temporal trend in prescribing was observed: the proportion of patients discharged on SAPT was 10.1% in the pre-2020 era (45 of 447) and 47.4% in the 2020–2023 era (102 of 215); the χ^2^ test for the era-by-regimen association was highly significant (χ^2^(1) = 117.4, *p* < 0.001).

### 3.3. Antiplatelet Regimen Transitions 

A documented change in antiplatelet regimen occurred in 88 of 662 patients (13.3%) during follow-up; the frequency, timing, direction, and recorded triggers of these transitions are summarized in Table 2. Switching was strongly asymmetric: 83 of 515 baseline-DAPT patients (16.1%) had a regimen change, of which the majority (71 of 83, 85.5%) was de-escalation to SAPT; a further 10 (12.0%) transitioned to a regimen incorporating oral anticoagulation (most commonly for new-onset atrial fibrillation). In contrast, only 5 of 147 baseline-SAPT patients (3.4%) had any modification, none of which represented escalation to DAPT. The median time from TAVI to a documented change was 19.6 months (IQR 9.9–45.3); 71.5% of all switches occurred after 12 months and 42.0% after 24 months. The most common driver other than routine de-escalation (n = 73) was new-onset atrial fibrillation requiring oral anticoagulation (n = 7); no documented regimen change in the cohort was preceded by a major bleeding event.

### 3.4. Primary Efficacy Endpoint: Ischemic MACE

**Figure 1:**
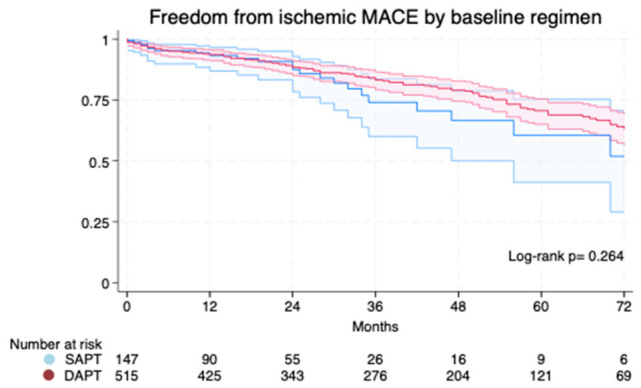
Intention-to-treat Kaplan–Meier estimates of freedom from ischemic MACE (death, stroke, or myocardial infarction) by baseline antiplatelet regimen at discharge. Log-rank *p* = 0.264. The Kaplan–Meier estimator is defined for a fixed exposure partition and therefore corresponds to the intention-to-treat sensitivity analysis.

During follow-up, 141 patients experienced an iMACE (24 SAPT, 117 DAPT). Kaplan–Meier estimates of freedom from iMACE by baseline regimen are shown in Figure 1; these curves correspond to the intention-to-treat sensitivity analysis rather than to the primary time-varying analysis. The accompanying log-rank test (*p* = 0.264) is correspondingly the ITT comparison. Hazard ratios from the full model hierarchy are summarized in Table 3. In the primary time-varying Cox model (with person-time attributed to the regimen actually being received), the adjusted hazard ratio for DAPT versus SAPT was 1.28 (95% CI 0.81–2.04; *p* = 0.292). None of the sensitivity estimates (intention-to-treat, 6-month landmark, or IPTW time-varying Cox) crossed conventional thresholds for statistical significance, and the 95% confidence intervals uniformly included the null. Because SAPT patients were concentrated in the contemporary era, procedural era (pre-2020 vs. 2020–2023) was added as a covariate to all adjusted models; the era-adjusted primary time-varying hazard ratio for DAPT versus SAPT was 1.25 (95% CI 0.78–2.00, *p* = 0.359), essentially unchanged from the original estimate, and the main effect of era itself was null (HR 1.01, 95% CI 0.56–1.82, *p* = 0.975). The era × regimen interaction was non-significant (Wald χ^2^(1) = 0.00, *p* = 0.967), with era-stratified hazard ratios of 1.25 (95% CI 0.75–2.10) for the pre-2020 era and 1.22 (95% CI 0.37–3.97) for the 2020–2023 era. The 2020–2023 stratum is small (13 events), and its confidence interval is correspondingly wide, but the directional consistency between strata and the formal interaction test argues against meaningful effect modification by era.

The proportional-hazards assumption was not violated. Absolute standardized mean differences (|SMD|) for baseline covariates before and after inverse-probability-of-treatment weighting are shown in Table S1. A threshold of 0.10 is conventionally considered an adequate balance. After weighting, all covariates achieved |SMD| ≤ 0.15, with all but two below the 0.10 threshold (Supplementary Table S1).

### 3.5. Net Clinical Benefit: NACE

**Figure 2:**
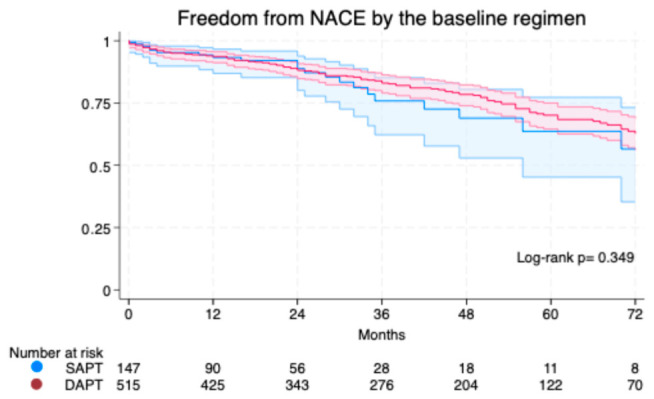
Intention-to-treat Kaplan–Meier estimates of freedom from NACE (death, stroke, myocardial infarction, or major bleeding) by baseline antiplatelet regimen at discharge. Log-rank *p* = 0.349. The curves correspond to the intention-to-treat sensitivity analysis.

NACE occurred in 146 patients (25 SAPT, 121 DAPT). The overall pattern mirrored that of iMACE, reflecting the small numerical contribution of bleeding to the composite in this anticoagulant-free cohort. Kaplan–Meier estimates of freedom from NACE by baseline regimen are shown in Figure 2; as for iMACE, these curves correspond to the intention-to-treat sensitivity analysis, and the accompanying log-rank test (*p* = 0.349) is the ITT comparison rather than a test of the time-varying framework. Hazard ratios for NACE are summarized in Table 4. In the primary time-varying Cox model, the adjusted hazard ratio for DAPT versus SAPT was 0.71 (95% CI 0.44–1.14; *p* = 0.159). As with iMACE, all sensitivity estimates were directionally consistent, and none reached conventional statistical significance.

### 3.6. Safety Endpoint: Major Bleeding

Major bleeding was rare in this anticoagulant-free cohort: 6 events occurred during follow-up (2 SAPT, 4 DAPT). The unadjusted Cox hazard ratio for DAPT versus SAPT was 0.64 (95% CI 0.09–4.62; *p* = 0.657). Bleeding point estimates derived from registry data should be interpreted with particular caution because major bleeding typically triggers de-escalation of antiplatelet therapy, a time-varying analysis of the bleeding endpoint is biased toward DAPT, and an intention-to-treat analysis is biased toward SAPT. The endpoint is therefore reported descriptively.

### 3.7. Stroke: 30-Day Landmark Analysis

A 30-day landmark separated peri-procedural from late strokes. Of 38 total strokes, 6 occurred within the first 30 days (1 SAPT, 5 DAPT) and 32 occurred thereafter (4 SAPT, 28 DAPT). In patients who were stroke-free at day 30, the unadjusted hazard ratio for late stroke with DAPT versus SAPT was 1.12 (95% CI 0.39–3.12; *p* = 0.831). These data provide no signal that maintenance antiplatelet regimen modulates the rate of late stroke in this population.

### 3.8. Individual Components

Cause-specific hazard ratios for each component of the composite endpoints are summarized in Table 5. All estimates are unadjusted, given the modest number of events for each component. Death was treated as a competing event for the three non-fatal components.

### 3.9. Sensitivity to Unmeasured Confounding (E-Values)

E-values for the primary and component endpoints are summarized in Supplementary Table S2. The E-value for the primary iMACE time-varying adjusted estimate (HR 1.28) was 1.66, indicating that an unmeasured confounder would have to be associated with both DAPT use and iMACE by a risk ratio of approximately 1.7, beyond every measured covariate, to fully explain the observed association. The corresponding E-values were 1.85 for NACE and 1.85 for the IPTW time-varying iMACE estimate. E-values for the cause-specific component HRs ranged from 1.43 (stroke) to 4.44 (major bleeding); the high E-value for bleeding reflects the imprecision of the small-event estimate rather than robustness and should not be interpreted as evidence of effect. The E-value for the confidence-interval limit closest to the null was 1.00 for every endpoint, because every confidence interval included the null value.

## 4. Discussion

The debate of SAPT vs. DAPT after different procedures is still ongoing [25,26,27]. In this contemporary single-center TAVI cohort of 662 patients without an indication for oral anticoagulation, analyses found no statistically significant difference between SAPT and DAPT for the efficacy composite of iMACE, the net composite of NACE, individual cardiovascular components, or late stroke beyond the peri-procedural window. Point estimates from the primary time-varying Cox model and from all sensitivity analyses were clustered close to the null, with confidence intervals that uniformly crossed unity. Major bleeding was rare (6 events in 662 patients) and any formal comparison between arms was therefore limited by imprecision rather than by a null effect.

The present cohort differs in some respects from contemporary Western TAVI populations. Patients were younger (median age ~75 years vs. ~80–84 in POPular TAVI, FRANCE-TAVI, and the STS/ACC TVT registry) [9,28,29], more often male (64% vs. approximately 50%) [9], and had a higher prevalence of diabetes mellitus compared to surgical patients [30]. Prescribing trends followed the global trajectory: SAPT use rose from approximately 10% in the pre-2020 era to 47% in the 2020–2023 era, broadly aligned with the post-POPular-TAVI shift documented in Western registries [9]. These demographic and prescribing differences do not affect internal validity but should be borne in mind when generalizing the findings to populations with a different age, sex, or comorbidity profile.

These observational findings are directionally consistent with the randomized evidence base. The POPular TAVI trial, which randomized 690 anticoagulant-free patients to aspirin alone versus aspirin plus clopidogrel, demonstrated a lower incidence of bleeding with SAPT and an ischemic event rate that was non-inferior to DAPT [9]. The ARTE trial and subsequent meta-analyses reached the same conclusion [10,13]. Our real-world data, collected outside trial enrolment windows, and containing the full spectrum of contemporary TAVI indications, extend these findings to an unselected population in which DAPT remained the more common discharge prescription for much of the study period, an important observation about the speed with which guideline recommendations translate into everyday practice [3,4].

A methodological observation deserves emphasis. The adjusted ITT Cox estimate for iMACE and the adjusted TV estimate differ in direction. Both confidence intervals include the null and overlap substantially, so the divergence does not formally demonstrate that DAPT-on patients experienced more ischemic events than SAPT-on patients. The directional difference is, however, methodologically informative and mechanistically explained by the transition pattern presented in Table 2. Eighty-five percent of all switches were DAPT-to-SAPT de-escalations at a median of 19.9 months. Thus, in the ITT framework, substantial person-time labeled as “DAPT” was actually spent on SAPT during the second through fifth post-TAVI years, when most events accumulated. The transition data also argue against event-driven regimen retention: the late timing of switches with a long preceding event-free interval, and the complete absence of SAPT-to-DAPT escalations or switches preceded by a documented ischemic or bleeding event, are inconsistent with clinicians retaining patients on DAPT in reaction to interim ischemic deterioration. Residual confounding by coronary-disease burden is partially addressed by adjustment for prior PCI and prior MI, by the E-value sensitivity analysis, and by the era-stratified analysis, although it cannot be definitively excluded.

A related but distinct methodological consideration is time-dependent confounding of the ischemic comparison. The same logic that motivates an ITT rather than a TV framework for the bleeding endpoint, that a clinical event may trigger regimen change, can in principle apply to ischemic outcomes. A standard time-varying Cox model with baseline covariates does not address this; the appropriate framework is a marginal structural model fitted with inverse-probability-of-treatment weights that are time-updated using the patient’s evolving covariate history [31]. Two features of our data partially mitigate the concern: 85.5% of all switches were DAPT-to-SAPT de-escalations with a long preceding event-free interval, and no documented switch was preceded by a major bleeding event, both arguing for protocolized rather than reactive switching; and the era-adjusted primary hazard ratio is essentially identical to the original estimate, suggesting that era-correlated determinants of switching are not driving the comparison. Neither argument fully excludes time-dependent confounding, however. We could not from the present registry construct the time-stamped longitudinal covariate history required to fit a marginal structural model, and we have therefore identified this as a methodological priority for future multi-center observational analyses of antiplatelet regimens after TAVI.

The bleeding endpoint in this cohort was numerically small. This reflects three factors: exclusion of anticoagulated patients, the conservative VARC-3 type 3b/4 definition, and a multi-channel ascertainment strategy. Despite multi-channel ascertainment, under-ascertainment of bleeding events managed at other hospitals or resolved without hospital admission cannot be definitively excluded, and the observed bleeding rate should not be interpreted as an absolute population-level estimate. The cohort is also underpowered to detect the bleeding advantage of SAPT consistently demonstrated in randomized trials and meta-analyses, and any inference about comparative safety from these six events would be speculative. We accordingly report the bleeding endpoint descriptively only, and the comparative-safety conclusion remains anchored to the randomized evidence rather than to the present data [9].

The 30-day stroke landmark analysis showed that stroke events were distributed between an early peri-procedural cluster (six strokes in the first 30 days) and a late cluster (32 strokes thereafter). Antiplatelet regimen had no detectable relationship with late stroke (HR 1.12, 95% CI 0.39–3.12), consistent with the predominantly embolic and procedure-related etiology of early stroke after TAVI and with the limited contribution of antiplatelet choice to the prevention of late cerebrovascular events in this population.

The principal strengths of this analysis are methodological. The primary estimator is biologically appropriate and computed on a properly structured counting-process dataset with cluster-robust standard errors; the sensitivity analyses cover the three most important sources of bias in observational antithrombotic studies (confounding by indication, immortal-time, and residual exposure misclassification). All outcomes were adjudicated against VARC-3 and Universal Definition criteria [17,18], and the cohort is broadly representative of contemporary TAVI practice, including valve-in-valve and transcaval procedures.

Several limitations should be acknowledged. First, this single-center observational cohort may have unmeasured confounding, particularly by frailty. Although IPTW balanced most measured covariates, residual imbalance persisted for prior PCI (|SMD| 0.146) and prior stroke (|SMD| 0.103, both markers of coronary/cerebrovascular disease burden and plausible confounders for ischemic outcomes. E-values for the confidence-interval limit were 1.00 throughout; thus, we do not claim equivalence between regimens, and IPTW estimates should be interpreted conditionally on the no-unmeasured-confounding assumption. Second, risk scores, fragility index, and procedural variables were not assessed, but are biologically plausible confounders. Era-stratified analysis partially mitigates this by absorbing era-correlated unmeasured procedural variables; however, residual confounding by procedural and frailty variables cannot be excluded.

Bleeding events managed elsewhere or without hospital contact cannot be definitively excluded. Accordingly, the NACE composite should be interpreted primarily as an ischemic-event statement, not a balanced safety-and-efficacy composite. The DAPT group was substantially larger than SAPT, reflecting practice patterns; analyses are well powered for the primary composite but less so for individual low-incidence components. Adherence to the discharge regimen beyond documented changes was not independently verified.

External validity is bounded by the cohort definition, patients without an indication for chronic oral anticoagulation, who constitute a large proportion of the contemporary TAVI population. Conclusions apply specifically to the SAPT-versus-DAPT decision in this subgroup and do not extend to TAVI patients with atrial fibrillation, venous thromboembolism, mechanical prosthesis, or other indications for chronic anticoagulation.

Finally, the primary time-varying Cox model with baseline-fixed covariates does not adjust for time-dependent confounders affected by prior treatment. Transition data argue against event-driven regimen change as a major mechanism, but residual time-dependent confounding cannot be definitively excluded.

Clinically, these data, interpreted in light of the residual imbalance for prior PCI and the E-value sensitivity analysis, are consistent with, but do not on their own establish, the contemporary guideline position that SAPT is a reasonable default antiplatelet strategy after TAVI in patients without another antithrombotic indication. They suggest that the absence of an ischemic-event penalty for SAPT, demonstrated in randomized trials, extends to unselected real-world practice, with the caveat that residual confounding by coronary-disease burden cannot be fully excluded. It should be noted that these clinical implications apply to TAVI patients without an indication for chronic OAC; for the substantial subgroup with an OAC indication, the relevant decision is between OAC monotherapy and OAC plus antiplatelet, and is guided by different evidence-base.

## 5. Conclusions

In a contemporary real-world cohort of TAVI patients without an indication for chronic oral anticoagulation, discharge antiplatelet regimen (SAPT vs. DAPT) was not associated with a statistically significant difference in ischemic MACE, NACE, or any individual cardiovascular component, across an analytic hierarchy comprising time-varying, intention-to-treat, landmark, and IPTW-weighted Cox models. The primary inference from these data is that SAPT does not carry an ischemic-event penalty compared with DAPT in real-world practice; the expected bleeding advantage of SAPT, demonstrated in randomized trials, could not be independently confirmed in this cohort owing to the small number of bleeding events. The findings are consistent with and provide observational support for the contemporary international valvular-disease guidelines, which favor SAPT as the default antiplatelet strategy after TAVI in patients without another indication for antithrombotic therapy.

## Figures and Tables

**Table 1:** Baseline characteristics of the primary cohort.

Variable	SAPT (n = 147)	DAPT (n = 515)	*p*
Age, years	74 (66–80)	76 (69–81)	0.033
BMI, kg/m^2^	28.5 (24.4–32.5)	29.4 (26.0–34.4)	0.005
Hemoglobin, g/dL	12.6 (10.9–14.1)	12.5 (11.2–13.8)	0.313
Platelets, ×10^9^/L	236 (186–287)	248 (199–294)	0.126
Creatinine, µmol/L	89 (74–120)	86 (72–108)	0.125
LVEF, %	55 (50–60)	55 (50–60)	0.710
Male sex	95/147 (64.6)	330/515 (64.1)	0.903
Prior cardiac surgery	15/130 (11.5)	46/501 (9.2)	0.418
Prior PCI	18/131 (13.7)	185/501 (36.9)	<0.001
Dialysis	8/139 (5.8)	16/502 (3.2)	0.158
Hypertension	104/139 (74.8)	401/504 (79.6)	0.228
Prior stroke	11/139 (7.9)	36/504 (7.1)	0.757
Chronic liver disease	6/128 (4.7)	11/368 (3.0)	0.399
Diabetes mellitus	94/139 (67.6)	341/505 (67.5)	0.982
COPD	18/139 (12.9)	83/504 (16.5)	0.313
Carotid stenosis	5/139 (3.6)	10/503 (2.0)	0.336
Home oxygen therapy	1/138 (0.7)	10/504 (2.0)	0.472
Prior MI	7/137 (5.1)	44/505 (8.7)	0.167
Pre-procedural PPM	3/131 (2.3)	10/501 (2.0)	0.738
Pre-procedural ICD	2/130 (1.5)	1/501 (0.2)	0.109
Previous aortic valve replacement	2 (1.4)	8 (1.6)	>0.99

Continuous variables: median (IQR), Mann–Whitney U test. Categorical variables: n/N (%), χ^2^ or Fisher’s exact test as appropriate. BMI, body-mass index; COPD, chronic obstructive pulmonary disease; ICD, implantable cardioverter-defibrillator; IQR, interquartile range; LVEF, left-ventricular ejection fraction; MI, myocardial infarction; PCI, percutaneous coronary intervention; PPM, permanent pacemaker. Per-variable missingness is reflected in the row-specific denominators; the difference between the row denominator and the group total (147 SAPT, 515 DAPT, 662 overall) corresponds to the number of patients with missing data for that variable.

**Table 2:** Antiplatelet regimen transitions during follow-up.

	n (%)
Patients with a documented regimen change	88/662 (13.3)
From baseline DAPT (n = 515)	83 (16.1)
From baseline SAPT (n = 147)	5 (3.4)
Direction of change—baseline DAPT switchers (n = 83)	
DAPT to SAPT (de-escalation)	71 (85.5)
DAPT to SAPT + oral anticoagulant	10 (12.0)
Within-DAPT drug substitution	2 (2.4)
Direction of change—baseline SAPT switchers (n = 5)	
Within-SAPT modification or addition of OAC	5 (100)
SAPT to DAPT (escalation)	0 (0)
Time from TAVI to documented change, months, median (IQR)	
All switchers (n = 88)	19.6 (9.9–45.3)
Baseline-DAPT switchers (n = 83)	19.9 (11.9–45.6)

**Table 3:** Hazard ratios for ischemic MACE, DAPT versus SAPT (SAPT = reference).

Model	HR (95% CI)	*p*
Intention-to-treat Cox, unadjusted	0.77 (0.49–1.22)	0.266
Intention-to-treat Cox, adjusted ^1^	0.66 (0.40–1.06)	0.087
Time-varying Cox, unadjusted	1.31 (0.85–2.03)	0.226
Time-varying Cox, adjusted ^1^ [PRIMARY]	1.28 (0.81–2.04)	0.292
6-month landmark Cox, adjusted ^1^	0.76 (0.45–1.30)	0.322
IPTW-weighted time-varying Cox	0.71 (0.44–1.16)	0.170

^1^ Adjusted for age, sex, BMI, LVEF, hypertension, diabetes mellitus, prior myocardial infarction, prior stroke, prior percutaneous coronary intervention, chronic lung disease, and chronic kidney disease. Global proportional-hazards test (primary model) *p* = 0.443. CI, confidence interval; HR, hazard ratio; IPTW, inverse-probability-of-treatment weighting.

**Table 4:** Hazard ratios for NACE, DAPT versus SAPT (SAPT = reference).

Model	HR (95% CI)	*p*
Intention-to-treat Cox, unadjusted	0.81 (0.52–1.26)	0.351
Intention-to-treat Cox, adjusted ^1^	0.70 (0.43–1.33)	0.146
Time-varying Cox, unadjusted	0.82 (0.53–1.25)	0.354
Time-varying Cox, adjusted ^1^ [PRIMARY]	0.71 (0.44–1.14)	0.159
6-month landmark Cox, adjusted ^1^	0.82 (0.49–1.40)	0.483

^1^ Adjusted for age, sex, BMI, LVEF, hypertension, diabetes mellitus, prior myocardial infarction, prior stroke, prior percutaneous coronary intervention, chronic lung disease, and chronic kidney disease. Global proportional-hazards test (primary model) *p* = 0.999.

**Table 5:** Cause-specific unadjusted hazard ratios for individual components (SAPT = reference).

Endpoint	Events (Total)	Cause-Specific HR (95% CI)	*p*
All-cause death	109	0.72 (0.43–1.22)	0.222
Stroke	38	0.91 (0.35–2.36)	0.851
Myocardial infarction	18	0.68 (0.22–2.08)	0.504
Major bleeding	6	0.40 (0.08–2.15)	0.287

Stroke, myocardial infarction, and major bleeding are modeled with death as a competing event.

## Data Availability

Data sharing needs the approval of the institution and signing data sharing agreement.

## Supplementary Materials

The following supporting information can be downloaded at: https://www.mdpi.com/article/10.3390/jcm15114381/s1, Table S1. Absolute standardized mean differences before and after IPTW; Table S2. E-values for the primary and component endpoints.

## Abbreviations

ACC	American College of Cardiology
AHA	American Heart Association
BMI	Body-mass index
CI	Confidence interval
COPD	Chronic obstructive pulmonary disease
DAPT	Dual antiplatelet therapy
DOAC	Direct oral anticoagulant
ESC	European Society of Cardiology
EACTS	European Association for Cardio-Thoracic Surgery
HR	Hazard ratio
ICD	Implantable cardioverter-defibrillator
iMACE	Ischemic major adverse cardiovascular events
IQR	Interquartile range
IPTW	Inverse probability of treatment weighting
LVEF	Left-ventricular ejection fraction
MI	Myocardial infarction
NACE	Net adverse clinical events
OAC	Oral anticoagulation
PCI	Percutaneous coronary intervention
PPM	Permanent pacemaker
SAPT	Single antiplatelet therapy
SMD	Standardized mean difference
STROBE	Strengthening the Reporting of Observational Studies in Epidemiology
TAVI	Transcatheter aortic valve implantation
VARC-3	Valve Academic Research Consortium 3
