# Supplementary material for: Single Versus Dual Antiplatelet Therapy After Transcatheter Aortic Valve Implantation in Patients Without Chronic Anticoagulation
Source: J Clin Med. 2026 Jun 5;15(11):4381. doi: 10.3390/jcm15114381 (PMC13258166; doi:10.3390/jcm15114381)
Supplement: Supplementary file 1 [file jcm-15-04381-s001.zip › jcm-4336916-supplementary.pdf]

Supplement:

Table S1. Absolute standardized mean differences before and after IPTW.

Covariate	|SMD| unweighted	|SMD| weighted
Age	0.194	0.067
Male sex	0.011	0.010
BMI	0.348	0.072
LVEF	0.018	0.038
Hypertension	0.112	0.040
Diabetes mellitus	0.002	0.014
Prior MI	0.142	0.076
Prior stroke	0.029	0.103
Prior PCI	0.554	0.146
Chronic lung disease	0.099	0.092
Chronic kidney disease	0.161	0.046

BMI, body-mass index; LVEF, left-ventricular ejection fraction; MI, myocardial infarction; PCI, percutaneous coronary intervention; SMD, standardized mean difference. Stabilized inverse-probability-of-treatment weights were computed as pT/ps for patients receiving DAPT and (1 − pT)/(1 − ps) for patients receiving SAPT, where ps is the predicted probability of DAPT from the propensity-score logistic model and pT is the marginal proportion receiving DAPT. Untrimmed stabilized weights had a mean of 1.00 (target 1.0), standard deviation of 0.35, and range of 0.40 to 5.12. One patient (0.2%) had a weight greater than 5, and no patient exceeded a weight of 10. The 1st and 99th percentiles, used as the trimming bounds, were 0.45 and 2.46, after which the weight range was 0.45 to 2.46. Propensity scores ranged from 0.51 to 0.98 with full overlap between the SAPT (0.52 – 0.96) and DAPT (0.51 – 0.98) groups, satisfying the positivity assumption. The propensity-score model was a logistic regression of DAPT versus SAPT on the pre-specified baseline covariates listed in the Methods.

Table S2: E-values for the primary and component endpoints.

Endpoint	HR (95% CI)	E-value, point	E-value, CI limit
iMACE, TV-Cox adjusted (primary)	1.28 (0.81–2.04)	1.66	1.00
NACE, TV-Cox adjusted	0.71 (0.44–1.14)	1.85	1.00
iMACE, IPTW TV-Cox	0.71 (0.44–1.16)	1.85	1.00
All-cause mortality (cause-specific)	0.72 (0.43–1.22)	1.82	1.00
Stroke (cause-specific)	0.91 (0.35–2.36)	1.43	1.00
Myocardial infarction (cause-specific)	0.68 (0.22–2.08)	2.30	1.00
Major bleeding (cause-specific)	0.40 (0.08–2.15)	4.44	1.00
Late stroke (>30 d landmark)	1.12 (0.39–3.12)	1.49	1.00
